# Magnolol mitigates morphine-induced analgesic tolerance via EGFR-mediated inhibition of microglial activation and neuroinflammatory reduction

**DOI:** 10.1016/j.gendis.2025.101802

**Published:** 2025-08-15

**Authors:** Weixin Lin, Yuxuan Wang, Qisheng Wang, Jiamin Huang, Ziting Zhou, Yongwei Jiang, Fenfen Qin, Zhonghao Li, Hui Wang, Zijing Wang, Haotian Pan, Qian Wang, Shanzhong Tan, Zhigang Lu

**Affiliations:** a Department of Integrated TCM and Western Medicine, Nanjing Hospital Affiliated to Nanjing University of Chinese Medicine, Nanjing, Jiangsu 210023, China; b School of Integrative Medicine, Nanjing University of Chinese Medicine, Nanjing, Jiangsu 210023, China; c Key Laboratory of Acupuncture and Medicine Research of Ministry of Education, Nanjing University of Chinese Medicine, Nanjing, Jiangsu 210023, China; d College of Pharmacy, Nanjing University of Chinese Medicine, Nanjing, Jiangsu 210023, China; e Institute of International Education, Nanjing University of Chinese Medicine, Nanjing, Jiangsu 210023, China

Opioid analgesics, including morphine, pose significant challenges in clinical pain management due to the development of tolerance—a phenomenon that necessitates escalating dosages to maintain analgesic efficacy while increasing risks of adverse effects and addiction.^1^ The mechanisms driving morphine tolerance involve neuroadaptive changes, particularly microglial activation within the pain modulatory circuitry spanning the periaqueductal gray (PAG)-rostral ventromedial medulla (RVM)-dorsal root ganglion (DRG).^2^^,^^3^ Notably, morphine-induced microglial activation has been shown to involve the epidermal growth factor receptor (EGFR) signaling pathway. Upon EGFR activation, microglia release pro-inflammatory cytokines that amplify nociceptive signaling, thereby critically contributing to tolerance progression.^4^ Emerging evidence suggests that bioactive components of *Cortex Magnoliae Officinalis* (*Houpo*), such as magnolol, effectively alleviate opioid withdrawal symptoms.^5^ Based on these findings, the present study aims to investigate the therapeutic potential of magnolol in mitigating chronic morphine tolerance and delineate its molecular mechanisms of action, with the ultimate goal of informing novel strategies for managing opioid tolerance.

In this study, we combined systematic network pharmacology, molecular docking, and molecular dynamics simulations to investigate the mechanism by which magnolol alleviates morphine tolerance, with experimental validation corroborating the computational findings. Through network integration (Fig. S1A–S1D), protein–protein interaction (PPI) network analysis (Fig. 1A) demonstrated that magnolol modulated 51 key nodes involved in morphine tolerance. Molecular docking of MCODE-derived core targets indicated strong binding affinity between magnolol and EGFR (binding energy: −7.2 kcal/mol) (Fig. 1B, C). Molecular dynamics simulations further revealed that the EGFR–magnolol complex achieved structural equilibrium (Fig. 1D; Fig. S2), as evidenced by the following: Root mean square deviation (RMSD) stabilization at approximately 3.8 Å, confirming system stability; Minor fluctuations in radius of gyration (Rg) and solvent-accessible surface area (SASA), suggesting localized conformational adjustments within an overall stable binding interface. These data suggest that magnolol has a stable and dynamic interaction with EGFR. Considering the critical role that EGFR plays in morphine-induced microglial activation and analgesic tolerance, magnolol shows promise as a candidate compound for modulating morphine analgesic tolerance.

**Figure 1:**
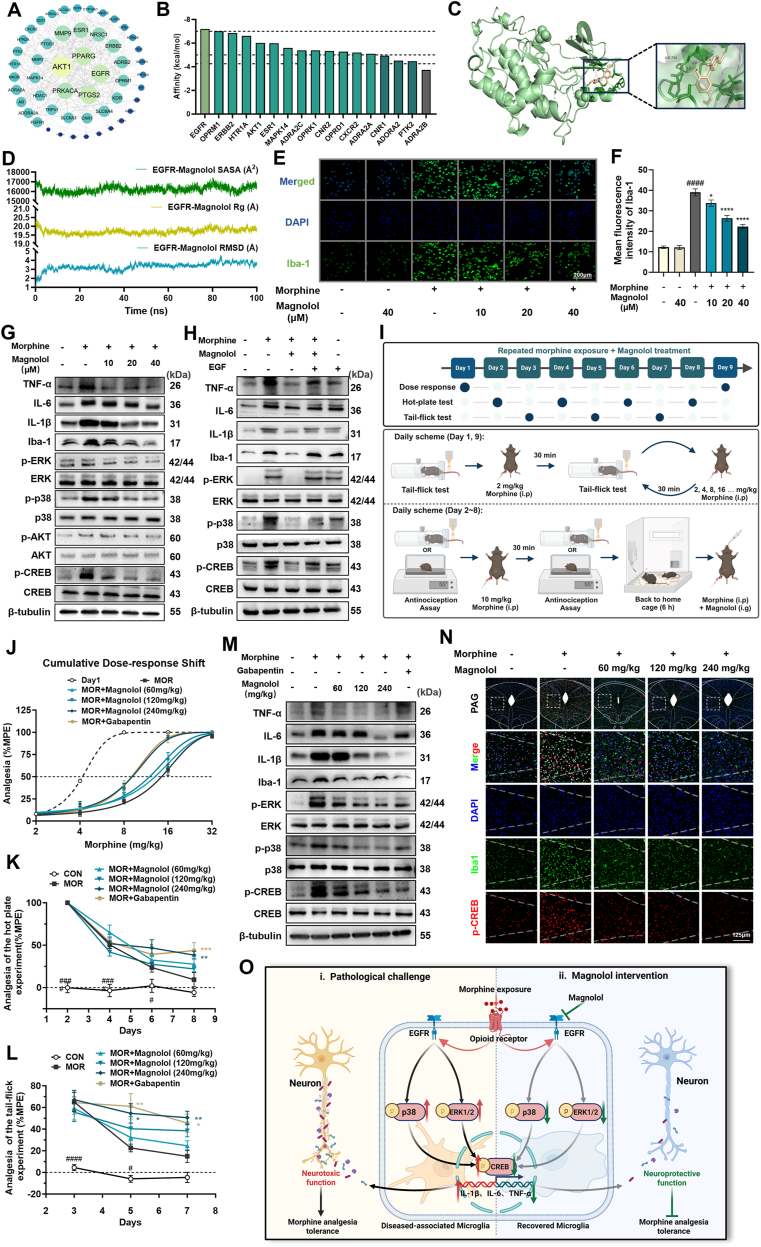
Magnolol attenuates morphine-induced analgesic tolerance through EGFR-mediated suppression of microglial activation and neuroinflammatory signalling. **(A)** Magnolol modulates the PPI network associated with morphine tolerance. **(B)** Molecular docking binding energy profiles of magnolol against core targets. **(C)** Molecular docking schematic of magnolol-EGFR interactions. **(D)** Molecular dynamics simulation trajectories of the magnolol-EGFR complex. **(E, F)** Immunofluorescence analysis and quantification of the microglial activation marker Iba-1 in morphine-treated BV2 cells with magnolol intervention (scale bar = 200 μm; *n* = 4), ^####^*P* < 0.0001 *vs* CON; ∗∗∗∗*P* < 0.0001, ∗∗∗*P* < 0.001, ∗*P* < 0.05 *vs* MOR, analyzed by one-way ANOVA with Dunnett's *post hoc* test. **(G)** Western blot analysis of pro-inflammatory cytokines (TNF-α, IL-6, and IL-1β), Iba-1 expression, and total/phosphorylated protein levels of ERK, p38, AKT, and CREB in BV2 cells after magnolol treatment. **(H)** Western blot analysis of pro-inflammatory cytokines (TNF-α, IL-6, and IL-1β), Iba-1 expression, and total/phosphorylated forms of ERK, p38, and CREB in BV2 cells. **(I)** Schematic of the experimental protocol for inducing morphine analgesic tolerance through chronic administration in mice. **(J**–**L)** The dose–response relationship between the measurement on Day 9 and the baseline measurement on Day 1 (*n* = 8–10). Changes in the percentage of MPE in the hot-plate test and tail-flick test from Day 2 to Day 8 (*n* = 8–10), ^####^*P* < 0.0001, ^#^*P* < 0.05 *vs* CON; ∗∗∗*P* < 0.001, ∗∗*P* < 0.01, ∗*P* < 0.05 *vs* MOR, were analyzed by two-way repeated measures ANOVA with Dunnett's multiple comparisons test. **(M)** Western blot analysis of pro-inflammatory cytokines (TNF-α, IL-6, and IL-1β), Iba1, and total/phosphorylated protein levels of ERK, p38 and CREB in PAG. **(N)** Representative immunofluorescence micrographs of p-CREB^+^ microglial cells and Iba1-related indicators in PAG. **(O)** Morphine-mediated analgesic tolerance pathways and mechanisms of Magnolol's blockade. Under pathological conditions, morphine induces the activation of opioid receptors in microglia, which facilitates EGFR dimerization and subsequent activation. This process initiates hyperphosphorylation of both the p38-MAPK and ERK-MAPK signalling pathways, ultimately leading to nuclear CREB activation and up-regulation of pro-inflammatory mediators (IL-6, IL-1β, and TNF-α). These cytokines mediate neurotoxic effects and neuronal hyperexcitability, thereby contributing to the development of morphine-induced analgesic tolerance. Upon magnolol administration, this compound specifically binds to EGFR in microglia, effectively blocking its dimerization process. Consequently, magnolol suppresses the p38/ERK-CREB pathway-mediated release of pro-inflammatory cytokines, attenuates neurotoxicity, restores normal neuronal excitability, and ultimately prevents the establishment of morphine tolerance.

To systematically assess microglial activation, we quantified the intensity of Iba-1 immunofluorescence in BV2 cells exposed to morphine. Maximal microglial activation, as evidenced by peak Iba-1 expression, was observed following 24-h treatment with 100 μM morphine (Fig. S3). The subsequent administration of magnolol (10, 20, and 40 μM) dose-dependently attenuated morphine-induced BV2 activation, significantly reducing Iba-1 levels compared to those in the morphine-treated group. Importantly, magnolol alone did not alter baseline Iba-1 expression or microglial activity under physiological conditions (Fig. 1E, F). Furthermore, magnolol treatment suppressed the morphine-triggered up-regulation of pro-inflammatory cytokines (TNF-α, IL-1β, and IL-6) and inhibited the phosphorylation of ERK, p38, and CREB (Fig. 1G; Fig. S4A), indicating its modulatory effects on neuroinflammatory signaling and EGFR-related pathways. To directly investigate the involvement of EGFR, we co-administered the EGFR agonist EGF (30 ng/mL) with magnolol. Notably, EGF treatment antagonized the magnolol-mediated suppression of Iba-1 and cytokine expression, concurrently restoring ERK, p38 and CREB phosphorylation to levels comparable to those of the morphine-treated controls (Fig. 1H; Fig. S4B). These results collectively demonstrate that magnolol mitigates morphine-induced microglial hyperactivation primarily through EGFR signaling blockade.

Furthermore, to evaluate the impact of magnolol on the development of morphine tolerance, we conducted tail-flick and hot-plate analgesic tests in mice (Fig. 1I). The baseline pain thresholds of the mice were stable, with no significant differences among groups (Fig. S5). Dose–response curves for morphine were established through percentage maximum possible effect (MPE) measurements in the tail-flick test on Days 1 and 9. Tolerance was induced by the intraperitoneal injection of morphine (10 mg/kg) twice daily from Day 2 to Day 8, and the MPE was measured 30 min later. The experimental groups received daily doses of magnolol (60, 120, or 240 mg/kg), while the positive control group was administered gabapentin (50 mg/kg). In MOR group mice, repeated administration significantly attenuated analgesic efficacy, as evidenced by reduced MPE values and rightward shifts in dose–response curves by Day 9. Co-administration of 240 mg/kg magnolol or 50 mg/kg gabapentin effectively preserved the analgesic potency of morphine, resulting in attenuated rightward curve shifts and increased MPE values compared to MOR group (Fig. 1J–L). These findings indicate that magnolol inhibits the development of morphine tolerance in a dose-dependent manner, with 240 mg/kg magnolol exhibiting efficacy comparable to that of the established positive control gabapentin.

Western blot (Fig. 1M; Fig. S6A) and immunofluorescence (Fig. 1N; Fig. S6B) analyses of the PAG revealed that magnolol treatment significantly attenuated morphine-induced microglial activation, as evidenced by reduced Iba-1 expression. Furthermore, magnolol exhibited dose-dependent suppression of pro-inflammatory cytokine levels (TNF-α, IL-1β, and IL-6) and inhibited the phosphorylation of ERK, p38, and CREB, the key downstream mediators of EGFR signaling.

This study demonstrated that magnolol attenuates morphine-induced analgesic tolerance through the EGFR-mediated modulation of microglial activity in the PAG. Mechanistically, magnolol suppresses p38/ERK-CREB signaling in PAG microglia, thereby reducing microglial activation and subsequent pro-inflammatory cytokine release. These neuroprotective pharmacological properties are associated with preserved morphine analgesic efficacy, as evidenced by behavioral assays. Our findings suggest that magnolol is a promising adjunctive therapy for mitigating opioid tolerance via targeted inhibition of neuroinflammatory pathways (Fig. 1O).

## CRediT authorship contribution statement

**Weixin Lin:** Writing – review & editing, Writing – original draft, Visualization, Validation, Supervision, Software, Resources, Project administration, Methodology, Investigation, Funding acquisition, Formal analysis, Data curation, Conceptualization. **Yuxuan Wang:** Writing – review & editing, Writing – original draft, Visualization, Validation, Supervision, Software, Resources, Project administration, Methodology, Investigation, Funding acquisition, Formal analysis, Data curation, Conceptualization. **Qisheng Wang:** Validation, Project administration, Methodology, Investigation, Funding acquisition. **Jiamin Huang:** Conceptualization. **Ziting Zhou:** Conceptualization. **Yongwei Jiang:** Software, Resources, Project administration. **Fenfen Qin:** Conceptualization. **Zhonghao Li:** Writing – original draft, Project administration. **Hui Wang:** Data curation. **Zijing Wang:** Software, Project administration, Conceptualization. **Haotian Pan:** Resources. **Qian Wang:** Conceptualization. **Shanzhong Tan:** Writing – review & editing, Writing – original draft, Investigation, Funding acquisition, Conceptualization. **Zhigang Lu:** Writing – original draft, Visualization, Validation, Supervision, Software, Resources, Project administration, Methodology, Investigation, Funding acquisition, Formal analysis, Data curation, Conceptualization.

## Ethics statement

All experiments were approved by the Committee on the Ethics of Laboratory Animal Experiments of Nanjing University of Chinese Medicine. Application form number: 202304A044.

## Funding

This work was supported by the National Key R&D Program of China (2024YFC3505405), National Natural Science Foundation of China (No. 82474341 and 82174498), Jiangsu Leading Talents in Traditional Chinese Medicine (SLJ0303), Jiangsu Province Acupuncture Moxibustion Integrated Education Ministry Key Laboratory Open Project (AML202306), the Subject of Academic Priority Discipline of Jiangsu Higher Education Institutions (2024) and National Administration of Traditional Chinese Medicine Youth Qihuang Scholars Support Project (2022).

## Conflict of interests

The authors declare no conflict of interests.
