# Supplementary material for: Magnolol mitigates morphine-induced analgesic tolerance via EGFR-mediated inhibition of microglial activation and neuroinflammatory reduction
Source: Genes Dis. 2025 Aug 15;13(1):101802. doi: 10.1016/j.gendis.2025.101802 (PMC12466129; doi:10.1016/j.gendis.2025.101802)
Supplement: Multimedia component 1 [file mmc1.docx]

**Material and Methods**

**Experimental cell line**

BV2 mouse microglia immortal cell line was presented by Hongyue Ma of Nanjing University of Chinese Medicine. Shape: Round or fusiform, short antennae or protrusions, adherent (both adherent and suspended) growth. Medium: High glucose medium DMEM containing 10% fetal bovine serum (FBS) and 1% penicillin-streptomycin. Incubator condition: 5% CO_2_, 37°C Temperature, 70%-80% humidity.

**Animals and Ethics statement**

Forty-six C57BL/6 adult male mice, weighing 22-24 g, were supplied by the Laboratory Animal Centre of Nanjing University of Traditional Chinese Medicine and placed in a lab with temperature and humidity control, with 12 h of light and 12 h of darkness. Food and water were supplied without charge. Prior to the experiments, the mice were divided randomly into different groups according to computer random numbering. Researchers conducting the experiments or analysing the data were not aware of the groups. All animal experimental protocols were in accordance with the ethical requirements of the Experimental Animal Ethics Committee of Nanjing University of Traditional Chinese Medicine (No. 202304A044, 2023-01-16).

**Preparation and administration of drugs for *in vitro* experiment**

Magnolol was purchased from Shanghai Yuanye Biotechnology (Article number: MFCD00016658, purity≥98%). Before each administration, weigh 10 mg magnolol (MW=266.33) powder and add 375.5 μL DMSO to prepare 100 mM solution which serves as the stock solution for use.

Morphine hydrochloride injection (181102-1) was bought from Jiangsu Province Pharmaceutical Co. LTD (Nanjing, China). Then add 1 mL morphine (MW = 285.34) hydrochloride injection to 0.33 μL HBSS to form 20 mM solution which serves as the stock solution for later use.

EGF was purchased from Target Molecule Corp. (Article number: TMPY-06984). Resuspend it with double-distilled water to a concentration of 1 mg/ml and the solution serves as the stock solution, and then dilute it with PBS.

BV2 cells in the logarithmic growth phase were seeded into poly-L-lysine-coated 96-well plates at a density of 2.5 × 10³ cells/well. After 24 hours of incubation, cells were treated with the respective compounds according to the experimental design.

**Preparation of drugs for *in vivo* experiment**

Magnolol was dissolved with corn oil for immediate use. Morphine solutions were freshly prepared with 0.9% saline to final concentration.

**Establishment of chronic morphine analgesic tolerance mouse model**

After a week of adaptive feeding, in addition to the CON group, the dose response test of morphine was carried out in the morning of Days 1 and 9 in the modeling period, and the doses of morphine were 2 mg/kg, 4 mg/kg, 8 mg/kg, 16 mg/kg and so on until the maximum possible effect (percent of the maximum possible effect, %MPE) was reached. MPE% = (effect time - baseline) / (maximum effect time - baseline) × 100%. On Days 2-8, all mice in the model and magnolol intervention groups were subjected to a long-term intervention of 10 mg/kg morphine twice daily for seven successive days. MPE was detected by hot plate method or heat radiation tail dump method 30 min after morning morphine administration.

Hot plate test: the mice were trapped on a plate at (55±0.5) °C, and the mice would show foot licking or jumping reaction after being stimulated by heat. The foot licking or jumping time (effect time) was recorded. In order to prevent damage to the mice, the maximum effect time was set at 30 s and MPE was calculated.

Tail flick test: the mice was placed in the fixator so that it could not move freely, and the mouse tail was naturally placed on the photothermal radiometer, and the parameter was set to 30 W. After the mouse was quiet, the time from the start of photothermal radiation to the swing of the mouse tail was recorded. In order to prevent injury in mice, regulation time is 16 s biggest effect, and calculate the MPE.

**Network pharmacological**

Firstly, magnolol targets were collected and predicted through TCMSP and SwissTargetPrediction database. Secondly, disease targets were obtained from Genecards, OMIM and DrugBank databases using keywords Opioid/Morphine tolerence. The collected magnolol target and disease target gene names were imported into VENNY 2.1 to obtain intersection targets and draw Venn maps.

The intersection target genes obtained from Draw Venn diagrams are uploaded to the Metascape platform, and the protein interaction network diagram combining the database relationships of STRING, BioGrid, OmniPath and InWeb_IM is obtained. Only the physical score of STRING and BioGrid were used for cluster analysis of the target, and the cluster analysis was carried out by MCODE algorithm to build the protein interaction (PPI) network diagram.

Finally, ensembl biomart online tool was used to obtain the ensembl number of all genes, uploaded to Omicshare platform, KEGG pathway analysis and GO enrichment analysis were selected, background genes were set as human genes, P-value mapping was selected for gene function enrichment and pathway enrichment analysis.

**Molecular docking**

Pubchem database and RCSB PDB database were used to search for chemical results of magnolol and macromolecular protein targets, and then openbabel software was used to convert SDF format into pdb format. autodock software was used to process the ligand magnolol and macromolecular protein targets, which were exported to pbdqt format. A semi-flexible docking method was used to combine the side chain of pocket amino acids and adjust the ligand conformation, and the binding free energy of the first 50 conformations was evaluated again by further optimization and GBVI/WSAdG method. The statistical optimal combination mode (the combination mode with the lowest free energy). Finally, the binding site of protein and ligand in the protein crystal structure was set as the binding pocket of the compound. The combinatorial pattern analysis diagram with the lowest free energy of intermolecular binding patterns was completed at PyMOL.

**molecular dynamics simulation**

A 100 ns molecular dynamics (MD) simulation of the complex was carried out using Gromacs 2023. The CHARMM 36 force field parameters were applied to the protein, and the ligand topology was constructed using the GAFF2 force field parameters. Periodic boundary conditions were employed, and the protein-ligand complex was placed in a cubic box. The TIP3P water model was used to fill the box with water molecules. The Particle Mesh Ewald (PME) method and the Verlet algorithm were used to handle the electrostatic interactions, respectively. Subsequently, 100,000 steps of equilibration in the isothermal-isochoric ensemble and the isothermal-isobaric ensemble were performed. The coupling constant was set to 0.1 ps, and the simulation lasted for 100 ps. Both the van der Waals and Coulomb interactions were calculated with a cutoff value of 1.0 nm. Finally, the system was subjected to a molecular dynamics simulation using Gromacs 2023 at a constant temperature of 300 K and a constant pressure of 1 bar for a total duration of 100 ns.

**Immunofluorescent staining**

The mice were anesthetized by intraperitoneal injection of 2% pentobarbital sodium, followed by normal saline and 4% paraformaldehyde solution. Then, whole brain fixation, dehydration and OCT embedding were performed. Finally, Leica thermostatic freezing microtome (-20°C) was used to freeze slices along the coronal plane of the whole brain of mice and cut into brain slices with a thickness of 8 μm.

In addition, *in vitro* experiments, microglia BV2 were transferred to a polylysine-treated cover glass 24-well plate with a cell count of 1×10^4^ cells/well and cultured to day 2 for drug treatment.

After getting slices of mouse brain tissue or a cell crawl, treatment with 3% H_2_O_2_ for 10min, the slides were incubated with PBS containing 5% BSA and 0.3% Triton X-100 for 1h. The primary antibody used was Iba-1 antibody (AF7143, 1:500, Beyotime). The secondary antibody was anti-Phospho-CREB antibody (9198, 1:500, Cell Signaling Technology). After 3 washes with PBS, sections were incubated with an Anti-rabbit IgG, HRP-linked Antibody (AFIHC023, 1:500, AiFang biological) at room temperature for 1 hour. The sections were then treated with a caseamide signal amplification kit (K1050-1051, APE×BIO). After 3 more washes, all slides were loaded with a medium containing DAPI (P0131, Beyotime). Images were collected by THUNDER Wide field High resolution imaging system and analyzed by ImageJ.

**Western blot**

Mouse brain tissue or BV2 cells were quickly collected on ice. Add RIPA protein lysate (containing 1% protease inhibitor and phosphatase inhibitor and 1% PMSF). The protein concentration was determined by BCA assay (P0010, Beyotime), and the loading buffer and PBS were used to configure a certain concentration of protein buffer. The samples were separated by 10% SDS-PAGE (20325ES62, Yeasen) and then transferred to PVDF membrane. The membrane was enclosed in 5% BSA at room temperature for 1 hour and incubated with the primary antibody at 4 °C overnight. After washing, cover with second antibody at room temperature for 50 minutes. Protein bands were detected with ECL detection reagent (E412-01, Vazyme) and imaged with chemical imaging system (5200CE, Tanon). Finally, the band intensities were totalled and analysed using Image J. Anti-IL-1 Beta antibody (16806-1-AP, 1:1000), anti-IL-6 antibody (21865-1-AP, 1:1000), anti-TNF Alpha antibody (17590-1-AP, 1: 1000), anti-p-38 MAPK antibody (14064-1-AP, 1:1000), anti-Phospho-p-38 antibody (28796-1-AP, 1:1000) were from Proteintech. Anti-AKT antibody (4685, 1:500), anti-Phospho-AKT antibody (4060, 1:500), Anti-Erk1/2 antibody (4695, 1:500), anti-Phospho-Erk1/2 antibody (4370, 1:500), Anti-CREB antibody (9197, 1:500), anti-Phospho-CREB antibody (9198, 1:500), and anti-β-tubulin antibody (2146, 1:1500) were from Cell Signaling Technology.


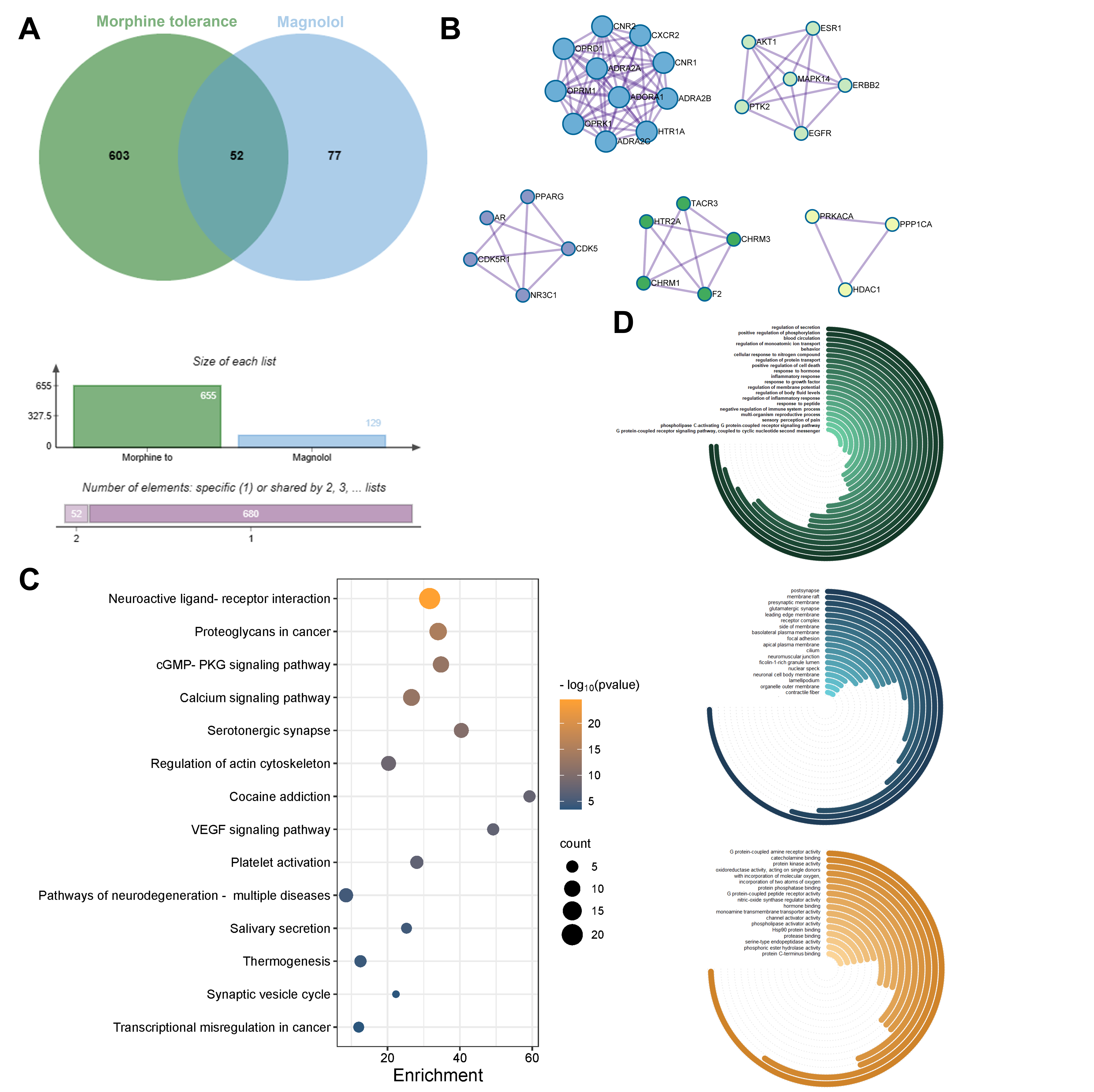


**Figure S1. Dissecting magnolol's impact on Opioid/Morphine tolerance via network pharmacology. (A)** Venn analysis of potential targets of magnolol intervention in opioid/morphine tolerance. **(B)** Analysis of MCODE, the core target of magnolol intervention in opioid/morphine tolerance. **(C)** Top 14 of KEGG Enrichment of magnolol intervention in opioid/morphine tolerance. **(D)** Analysis of the Top 20 Enriched GO Terms: Cellular Component (CC), Molecular Function (MF), and Biological Process (BP).


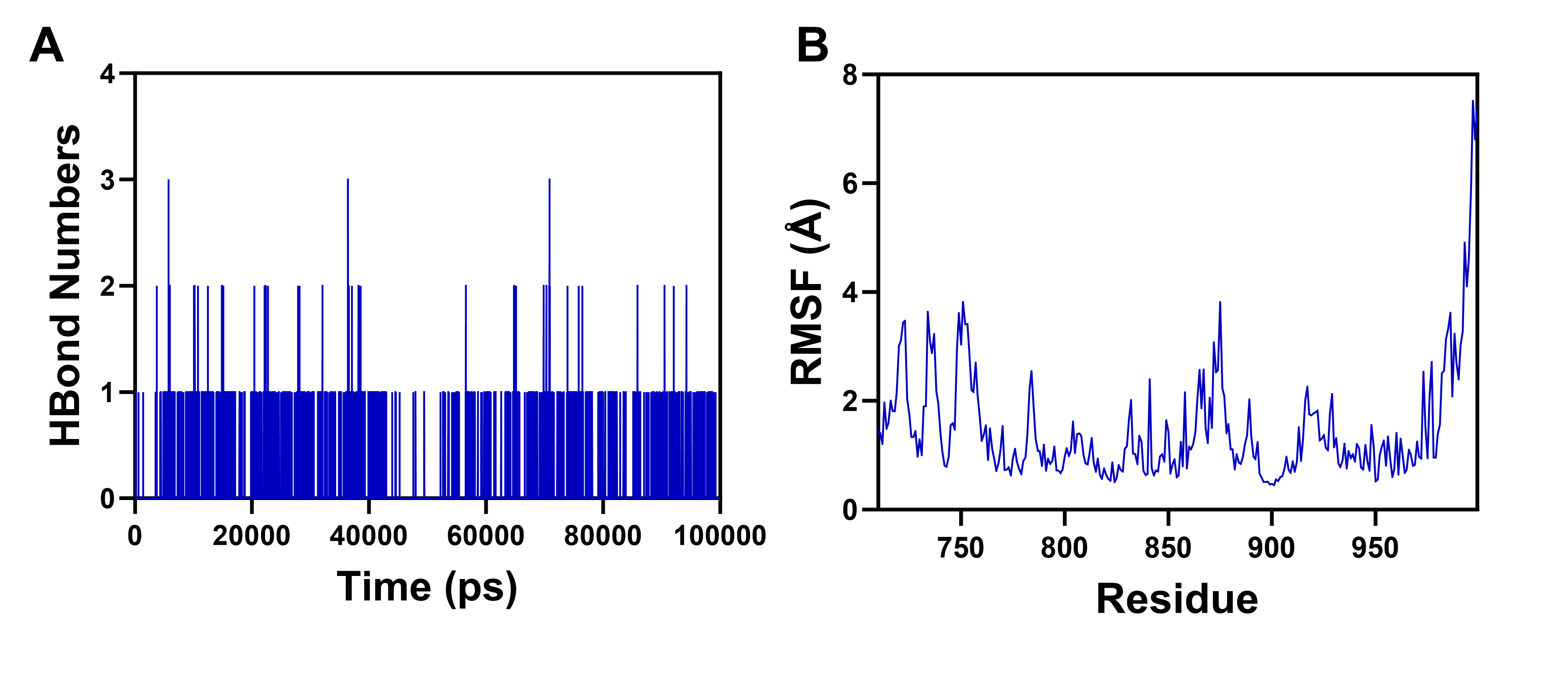


**Figure S2. Study of hydrogen bond changes and protein RMSF in EGFR-magnolol complex.** **(A)** Graph of the change in the number of hydrogen bonds during the kinetic process of the complex system of the ligand (Magnolol) and the target protein (EGFR). **(B)** Graph of the root mean square fluctuation (RMSF) of the protein in the EGFR-Magnolol complex system.


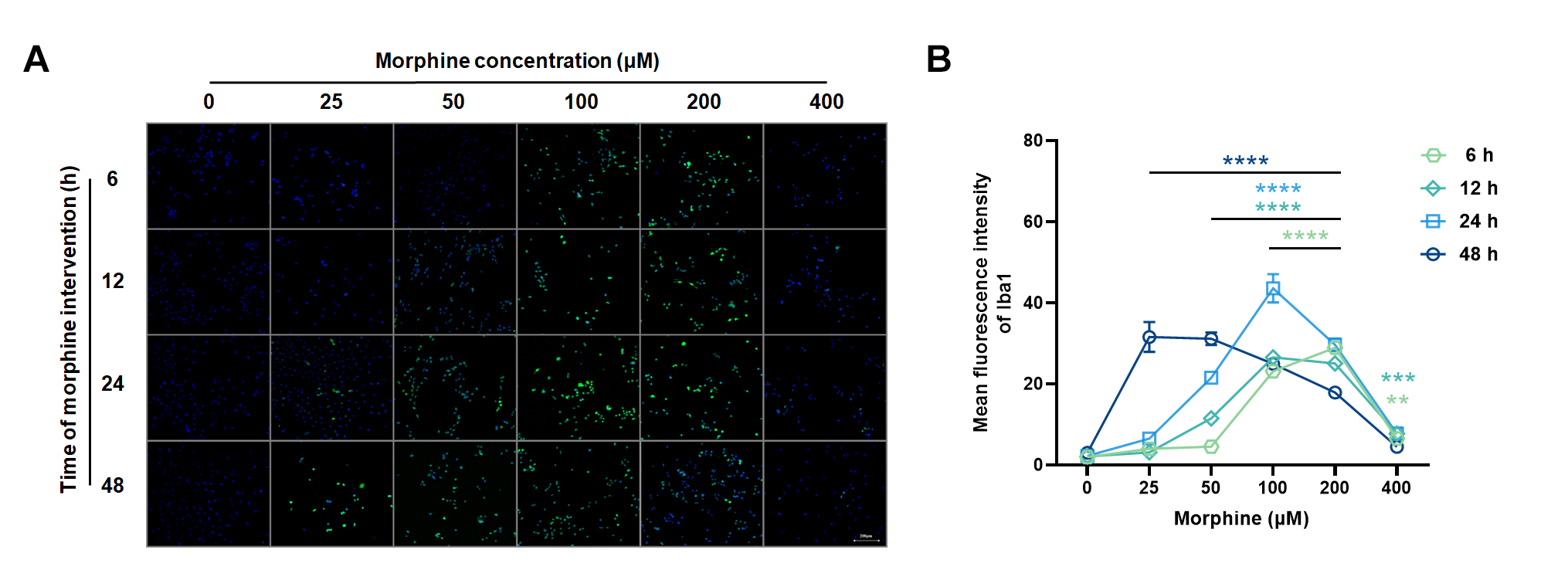


**Figure S3. Analysis of morphine's influence on Iba-1 in microglial activation.**

**(A)** Immunofluorescence map of induced microglial activation marker Iba-1 in chronic morphine treatment at different doses and times (scale: 200 μm). **(B)** Time and dose response of morphine to Iba-1 expression, n=4. All data were presented as mean ± SEM. One-way ANOVA was applied to test for group differences, compared to the average fluorescence intensity of 0 μM. ^****^*P* < 0.0001, ^***^*P* < 0.001, ^**^*P* < 0.01.


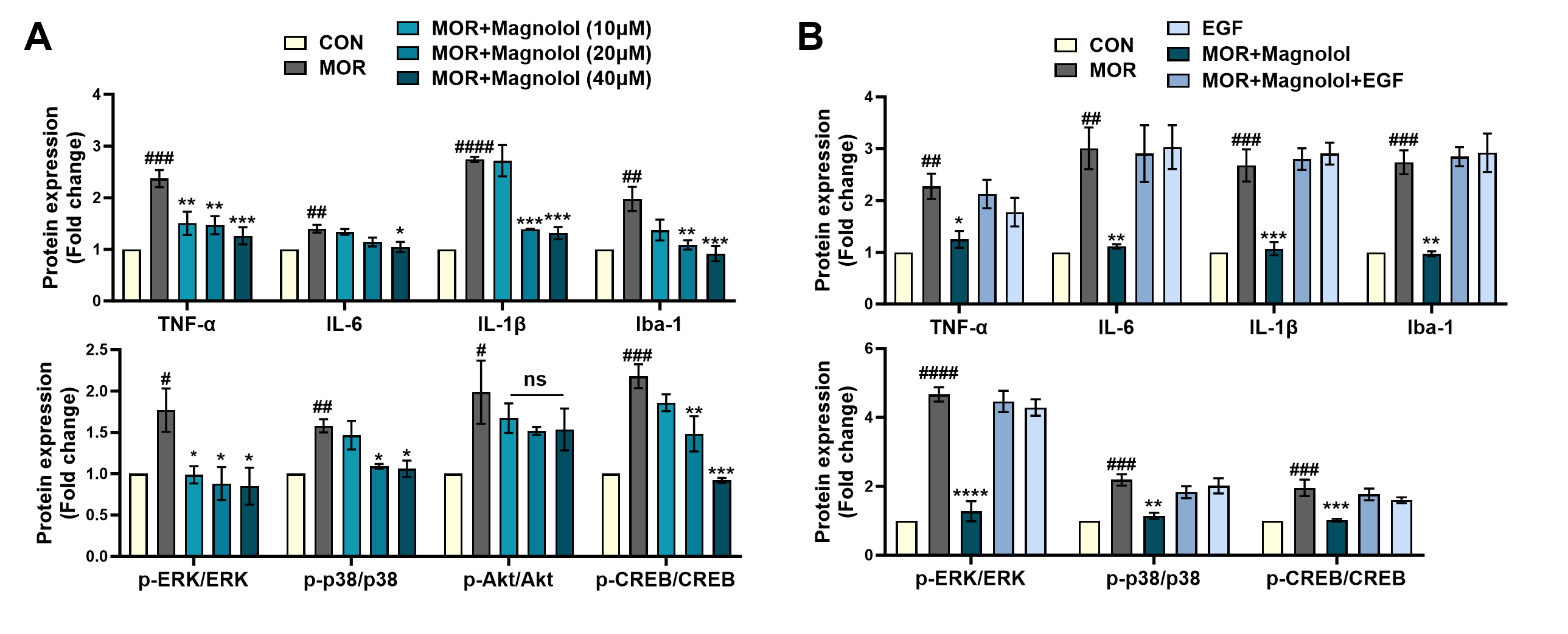


**Figure S4. Magnolol alleviates morphine-induced excessive activation of BV2 microglia by blocking EGFR signaling. (A)** Western blot quantification of pro-inflammatory cytokines (TNF-α, IL-6, and IL-1β), Iba-1 expression, and total/phosphorylated protein levels of ERK, p38, AKT, and CREB in BV2 cells after magnolol intervention (n = 3-4), ^####^*P* < 0.0001 ^###^*P* < 0.001, ^##^*P* < 0.01, ^#^*P* < 0.05 vs CON; ^***^*P* < 0.001, ^**^*P* < 0.01, ^*^*P* < 0.05 vs MOR, analyzed by one-way ANOVA with Dunnett's post hoc test. **(B)** Western blot quantification of pro-inflammatory cytokines (TNF-α, IL-6, IL-1β), Iba-1 expression, and total/phosphorylated forms of ERK, p38, and CREB in BV2 cells after EGF intervention (n = 3-4), ^####^*P* < 0.0001, ^###^*P* < 0.001, ^##^*P* < 0.01 vs CON; ^****^*P* < 0.0001, ^***^*P* < 0.001, ^**^*P* < 0.01, ^*^*P* < 0.05 vs MOR, analyzed by one-way ANOVA with Dunnett's post hoc test.


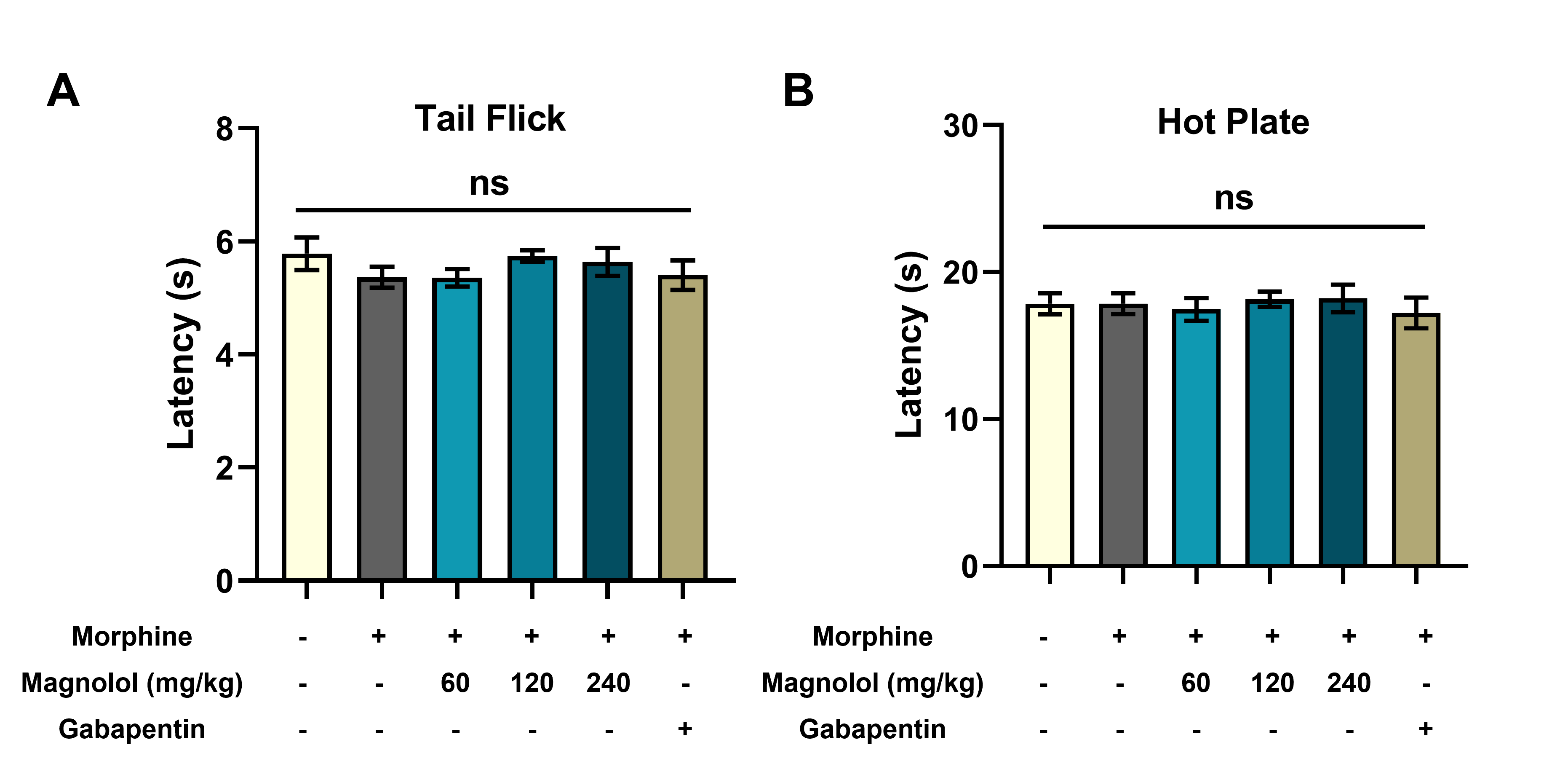


**Figure S5. MPE baseline values during pain tests. (A)** MPE baseline in hot plate test during the experiment, n=8-10. **(B)** MPE baseline in tail flick test during the experiment, n=8-10. All data were presented as mean ± SEM. One-way ANOVA was applied to test for group differences. "ns" represented *P* > 0.05.


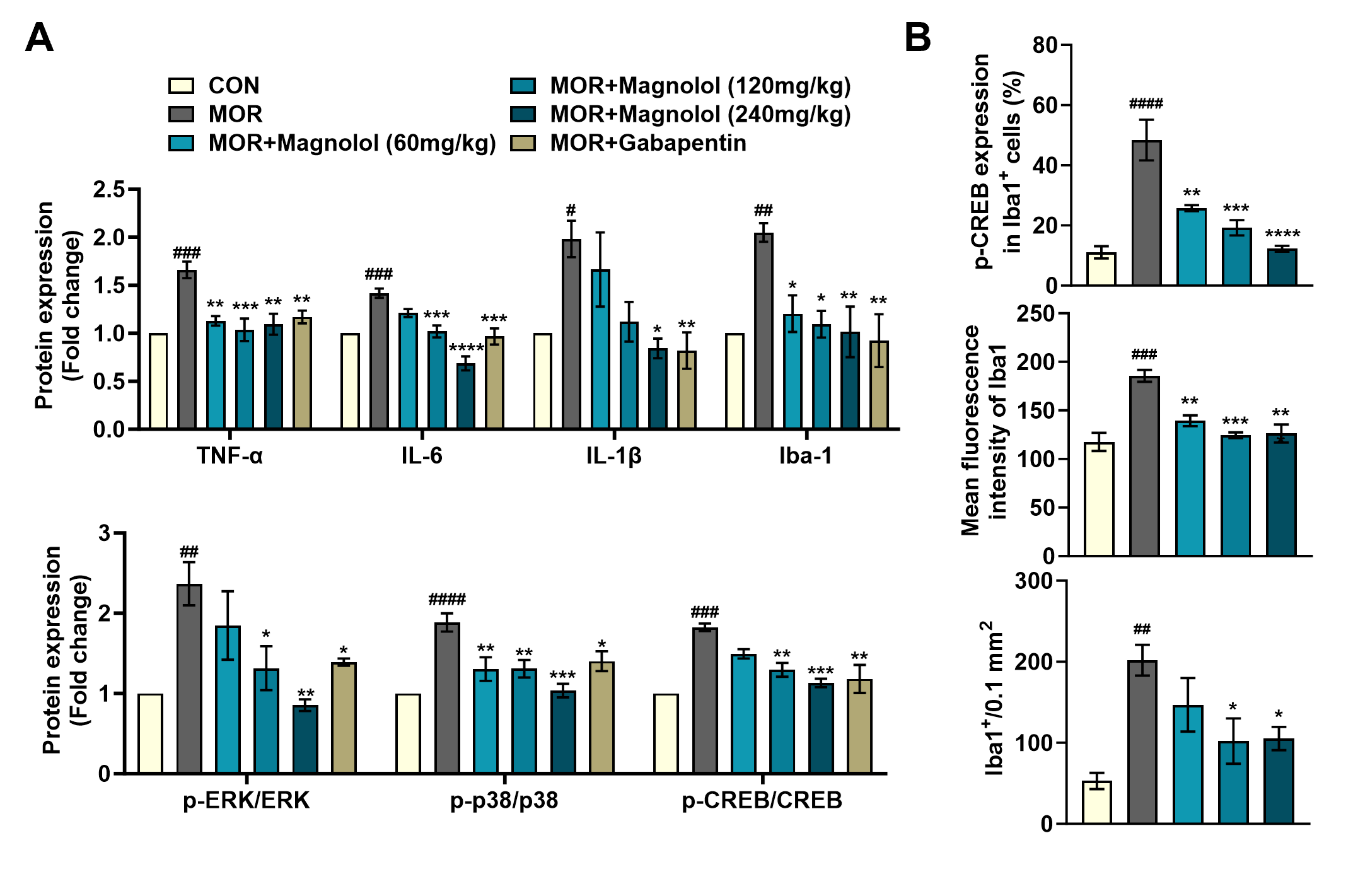


**Figure S6. Magnolol alleviates morphine-induced excessive activation of microglia and release of inflammatory factors by blocking the EGFR signaling pathway in PAG. (A)** Western blot quantification of pro-inflammatory cytokines (TNF-α, IL-6, and IL-1β), Iba1, and total/phosphorylated protein levels of ERK, p38, and CREB in PAG (n = 3), ^####^P < 0.0001, ^###^P < 0.001, ^##^P < 0.01, ^#^*P* < 0.05 VS CON; ^***^P < 0.001, ^**^P < 0.01, ^*^P < 0.05 VS MOR, analyzed by one-way ANOVA with Dunnett's post hoc test. **(B)** Quantification of p-CREB⁺ microglial cells in PAG, Iba1 mean fluorescence intensity and numerical density of Iba1⁺ cells per 0.1 mm² (scale bars = 125 μm; n = 3), ^####^*P* < 0.0001, ^###^*P* < 0.001, ^##^*P* < 0.01 vs CON; ^****^*P* < 0.0001, ^***^*P* < 0.001, ^**^*P* < 0.01, ^*^*P* < 0.05 vs MOR, analyzed by one-way ANOVA with Dunnett's post hoc test.
